# Transcription analysis of recombinant industrial and laboratory *Saccharomyces cerevisiae* strains reveals the molecular basis for fermentation of glucose and xylose

**DOI:** 10.1186/1475-2859-13-16

**Published:** 2014-01-28

**Authors:** Akinori Matsushika, Tetsuya Goshima, Tamotsu Hoshino

**Affiliations:** 1 Biomass Refinery Research Center (BRRC), National Institute of Advanced Industrial Science and Technology (AIST), 3-11-32 Kagamiyama, Higashi-Hiroshima, Hiroshima 739-0046, Japan; 2 Graduate School of Life Science, Hokkaido University, N10W8 Kita-ku, Sapporo, Hokkaido 060-0810, Japan

**Keywords:** Transcriptomics, DNA microarray, *Saccharomyces cerevisiae*, Xylose fermentation, Ethanol production

## Abstract

**Background:**

There has been much research on the bioconversion of xylose found in lignocellulosic biomass to ethanol by genetically engineered *Saccharomyces cerevisiae*. However, the rate of ethanol production from xylose in these xylose-utilizing yeast strains is quite low compared to their glucose fermentation. In this study, two diploid xylose-utilizing *S. cerevisiae* strains, the industrial strain MA-R4 and the laboratory strain MA-B4, were employed to investigate the differences between anaerobic fermentation of xylose and glucose, and general differences between recombinant yeast strains, through genome-wide transcription analysis.

**Results:**

In MA-R4, many genes related to ergosterol biosynthesis were expressed more highly with glucose than with xylose. Additionally, these ergosterol-related genes had higher transcript levels in MA-R4 than in MA-B4 during glucose fermentation. During xylose fermentation, several genes related to central metabolic pathways that typically increase during growth on non-fermentable carbon sources were expressed at higher levels in both strains. Xylose did not fully repress the genes encoding enzymes of the tricarboxylic acid and respiratory pathways, even under anaerobic conditions. In addition, several genes involved in spore wall metabolism and the uptake of ammonium, which are closely related to the starvation response, and many stress-responsive genes mediated by Msn2/4p, as well as trehalose synthase genes, increased in expression when fermenting with xylose, irrespective of the yeast strain. We further observed that transcript levels of genes involved in xylose metabolism, membrane transport functions, and ATP synthesis were higher in MA-R4 than in MA-B4 when strains were fermented with glucose or xylose.

**Conclusions:**

Our transcriptomic approach revealed the molecular events underlying the response to xylose or glucose and differences between MA-R4 and MA-B4. Xylose-utilizing *S. cerevisiae* strains may recognize xylose as a non-fermentable carbon source, which induces a starvation response and adaptation to oxidative stress, resulting in the increased expression of stress-response genes.

## Background

Interest in renewable energy sources is increasing substantially as an alternative to conventional fossil energy. Ethanol biofuel produced from lignocellulosic biomass generated in the agricultural and forestry sectors is a promising renewable energy source [1]. The yeast *Saccharomyces cerevisiae* is the preferred organism for industrial ethanol production from sugar derived from starch and sucrose due to its high growth rate, rapid fermentation rate, and high ethanol productivity under anaerobic conditions, together with high tolerance for ethanol and low pH. However, sugar derived from lignocellulosic biomass is a mixture of hexoses (primarily glucose) and pentoses (primarily xylose), and native strains of *S. cerevisiae* are unable to ferment xylose. Numerous studies have been conducted to develop metabolically engineered *S. cerevisiae* strains capable of utilizing xylose for ethanol production [2-6].

As *S. cerevisiae* is only able to metabolize xylulose, an isomerization product of xylose, the conversion of xylose to xylulose is crucial for the metabolic engineering of an efficient xylose-utilizing *S. cerevisiae* strain. Anaerobic xylose fermentation by *S. cerevisiae* was first demonstrated by heterologous expression of *XYL1* and *XYL2* genes encoding xylose reductase (XR) and xylitol dehydrogenase (XDH) from the yeast *Scheffersomyces* (*Pichia*) *stipitis*[7,8]. However, the resulting strains produce a considerable amount of xylitol as a by-product, thereby decreasing ethanol yields, mainly due to the difference in coenzyme specificities between XR (with NADPH) and XDH (with NAD^+^), which creates an intracellular redox imbalance [9]. Although heterologous expression of fungal or bacterial xylose isomerase (XI) that can directly convert xylose to xylulose is one solution to avoid cofactor imbalance in *S. cerevisiae*[10-13], many XI activities are too low to enable anaerobic growth on xylose, and the rate of xylose consumption is much lower in the XI-expressing *S. cerevisiae* strains than in the XR- and XDH-expressing strains [14,15]. However, some recombinant XI-expressing *S. cerevisiae* strains that ferment xylose to ethanol at high conversion rates have been reported [16-18]. In particular, Zhou et al. [17] recently reported that combining metabolic and evolutionary engineering generates new XI-expressing strains of *S. cerevisiae* with a greatly improved anaerobic growth rate (0.203 h^-1^), xylose conversion rate (1.866 g g^-1^ h^-1^), and ethanol yield (0.41 g/g).

Genome-wide analyses through synthetic genetic arrays, transcriptomics, proteomics, metabolomics, and fluxomics have been carried out to understand the genetic and physiological states of xylose metabolism [19-29]. For instance, we previously performed a comprehensive metabolome analysis using capillary electrophoresis time-of-flight mass spectrometry (CE-TOFMS) on the recombinant industrial *S. cerevisiae* strain MA-R4 during fermentation with different carbon sources, and demonstrated that low carbon flux through glycolysis from the PPP is one of the biggest factors restricting xylose utilization, and carbon and energy starvation conditions are induced in MA-R4 during fermentation with xylose [30]. Although these results provide a metabolic explanation for the low ethanol productivity on xylose compared to glucose, little is known about transcriptional differences between anaerobic glucose and xylose fermentation by MA-R4.

Industrial *S. cerevisiae* strains are generally superior ethanol producers in view of their inhibitory tolerance and high ethanol productivity compared to laboratory *S. cerevisiae* strains. For instance, industrial sake brewing yeasts have been selected for hundreds of years to have characteristics suitable for sake brewing, including high ethanol tolerance, high ethanol productivity, and high osmotic tolerance. To date, both laboratory and industrial *S. cerevisiae* strains have been metabolically engineered for improved xylose utilization [3]. In a previous study, we showed that the rates of aerobic xylose growth and anaerobic xylose fermentation of recombinant industrial strains, including MA-R4, were higher than those of recombinant laboratory strains [31]. In fermentations of mixed sugars containing glucose and xylose, the recombinant laboratory strains exhibited about a four-fold slower ethanol fermentation of both glucose and xylose as compared with the recombinant industrial strains [31]. Nevertheless, very little is known about the mechanisms responsible for the different characteristics between xylose-utilizing recombinant industrial and laboratory strains.

DNA microarray is a powerful tool to characterize differences in transcription levels as a function of cultivation conditions and strain differences, and to identify metabolic targets for the improvement of the rate and yield of ethanol production from xylose. Here, we carried out global transcriptional analysis based on DNA microarray to evaluate the differences in fermentation between the recombinant industrial strain, MA-R4, and the new recombinant laboratory strain, MA-B4, as well as the effects of two different carbon sources containing glucose and xylose. The aim of this study was to identify some of the genetic factors responsible for the high ethanol productivity on glucose compared to xylose, and for the high fermentation efficiency by industrial strains compared with laboratory strains. We identified certain genes and functional categories that are involved in ergosterol biosynthesis, central carbon metabolism, respiratory metabolism, hexose and other membrane transport systems, galactose metabolism, ATP synthesis, and response to starvation and stress.

## Results and discussion

### Anaerobic batch fermentations

**Figure 1:**
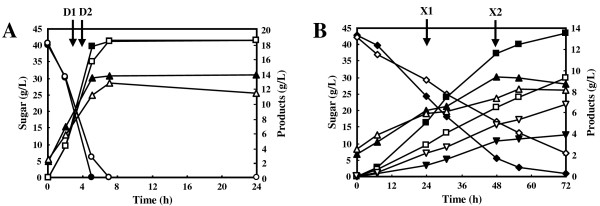
**Time-dependent batch fermentation profiles of glucose consumption (circles), xylose consumption (diamonds), ethanol production (squares), dry cell weight (triangles), and xylitol excretion (inverted triangles) by recombinant *S*. *cerevisiae*, MA-R4 (closed symbols) and MA-B4 (open symbols), in YPD medium containing 40 g/L glucose (A) and YPX medium containing 40 g/L xylose (B) under anaerobic conditions.** Small amounts of glycerol and acetic acid are not shown (see text). Data points represent the averages of three independent experiments. The arrows (denoted by D1, D2, X1, and *X*2) indicate times at which samples were taken for transcriptome analysis.

To determine the effects of different carbon sources (xylose and glucose) and different sources of yeast (industrial and laboratory strains) on ethanol fermentation, fermentation by the recombinant *S. cerevisiae* strains MA-R4 (industrial) and MA-B4 (laboratory) was performed anaerobically in YP-based media supplied with 40 g/L glucose (YPD medium) and 40 g/L xylose (YPX medium) in which aureobasidin A was not included. These strains exhibited stable recombinant enzyme activities and could be cultured in nonselective (YPD and YPX) media without significant loss of their xylose-fermenting ability for more than 10 generations (data not shown). In other words, these strains were stable and could grow on medium without aureobasidin A and without deletion of the integrated genes. As shown in Figure 1A and B, MA-R4 and MA-B4 showed different fermentation patterns depending on the carbon source of the medium. Strain MA-R4 grew anaerobically on glucose with a specific growth rate of 0.37 ± 0.01 h^-1^, whereas the growth rate of strain MA-B4 was 0.30 ± 0.02 h^-1^. When xylose was provided as the sole carbon source, MA-R4 grew at a higher growth rate than MA-B4, with growth rates of 0.031 ± 0.001 h^-1^ for MA-R4 and 0.020 ± 0.001 h^-1^ for MA-B4. Thus, the specific growth rate of MA-R4 was more than 1.2-1.5-fold higher than that of MA-B4 in both YPD and YPX media. After a 7.5-h fermentation in YPD medium, the cell concentrations of MA-R4 and MA-B4 reached 13.7 g/L DCW and 12.7 g/L DCW, respectively (Figure 1A). On the other hand, in YPX medium, the cell concentration of MA-R4 reached 9.35 g/L DCW after a 48-h fermentation, while that of MA-B4 reached 8.29 g/L DCW after a 56-h fermentation (Figure 1B). Biomass yields calculated from these cell concentrations of MA-R4 were 12-18% higher than those of MA-B4 in fermentation using YPD and YPX media. Thus, MA-R4 was apparently capable of utilizing complex media components slightly more efficiently for biomass and growth than MA-B4.

The rates of maximum specific glucose consumption and maximum specific ethanol production were similar in MA-R4 and MA-B4 in fermentation with YPD medium (Table 1). The maximum ethanol concentrations achieved by both MA-R4 and MA-B4 were approximately 18.6 g/L after a 7.5-h fermentation in YPD medium (Figure 1A). After 7.5 h of fermentation with YPD, the ethanol yield per gram of consumed glucose (g/g) by MA-R4 was identical (0.46 g/g: 89-90% of the theoretical yield) with that by MA-B4 (Table 1). Thus, it appears that both strains had almost identical ethanol production capabilities during glucose fermentation with respect to the maximum production rate, yield, and maximum titer of ethanol. Meanwhile, the specific xylose consumption rate was 31% higher in MA-R4 (0.12 g-xylose/g-DCW/h) than in MA-B4 (0.090 g-xylose/g-DCW/h) (Table 1). After 72 h of fermentation using YPX medium, MA-R4 metabolized almost all the xylose and produced 13.5 g/L of ethanol; however, xylose fermentation by MA-B4 was not completed within 72 h (Figure 1B). MA-R4 had a 36% higher rate of maximum specific ethanol production than MA-B4 in fermentation with YPX medium (Table 1). The maximum specific ethanol production rates of both strains achieved in fermentation with YPX were 91-93% lower than those with YPD (Table 1), which is consistent with our previous results from fermentation with glucose or xylose alone [30,32]. After 72 h of fermentation with YPX, the ethanol yield (0.34 g/g) of MA-R4 was higher than that (0.27 g/g) of MA-B4 (Table 1). The ethanol yields in YPX by MA-R4 and MA-B4 corresponded to 66 and 52% of the theoretical yield, respectively. Thus, the production rate and yield of ethanol from xylose is more strongly affected than that from glucose between the two recombinant strains.

**Table 1:** **Comparison of fermentation performance of *Sn cerevisiae *** **strains MA-R4 and MA-B4 in anaerobic batch fermentations with YPD and YPX media**

**Strain**	**Medium**	**Max. specific glucose consumption rate (g-glucose/g-DCW/h)**	**Max. specific xylose consumption rate (g-xylose/g-DCW/h)**	**Max. specific ethanol production rate (g-ethanol/g-DCW/h)**	**Biomass yield (g-DCW/g-consumed total sugar)**	**Ethanol yield (g-ethanol/g-consumed total sugar)**	**Xylitol yield (g-xylitol/g-consumed xylose)**	**Glycerol yield (g-glycerol/g-consumed total sugar)**	**Acetic acid yield (g-acetic acid/g-consumed total sugar)**
MA-R4	YPD	0.60 ± 0.06	ND	0.28 ± 0.02	0.29 ± 0.00	0.46 ± 0.01	ND	0.026 ± 0.001	0.010 ± 0.001
	YPX	ND	0.12 ± 0.01	0.026 ± 0.001	0.20 ± 0.00	0.34 ± 0.01	0.098 ± 0.001	0.077 ± 0.004	0.012 ± 0.001
MA-B4	YPD	0.58 ± 0.04	ND	0.26 ± 0.02	0.26 ± 0.01	0.46 ± 0.00	ND	0.019 ± 0.001	0.012 ± 0.000
	YPX	ND	0.09 ± 0.01	0.019 ± 0.001	0.17 ± 0.00	0.27 ± 0.01	0.20 ± 0.00	0.043 ± 0.001	0.011 ± 0.000

Values are the averages of three independent experiments ± standard deviation.

ND, not detectable.

In both strains, glycerol accumulation was maintained at a level lower than 1.1 g/L during glucose fermentation, and at a level lower than 3.1 g/L during xylose fermentation. A minimal amount of acetic acid (no more than 0.7 g/L) was produced mainly during glucose fermentation. After 72 h of fermentation with YPX, MA-R4 and MA-B4 excreted 3.94 and 6.81 g/L of xylitol, respectively (Figure 1B). Compared with MA-B4, MA-R4 showed a 42% decrease in xylitol excretion with a 45% increase in ethanol production. As shown in Table 1, the xylitol yield doubled from 0.098 g of xylitol/g of consumed xylose in MA-R4 to 0.20 g/g in MA-B4. The lower ethanol yield of MA-B4 was apparently directly related to its very high xylitol yield. Meanwhile, for both media, the glycerol yield was lower in MA-B4 than in MA-R4 (Table 1). The acetic acid yield of both strains was relatively constant during fermentation using YPD and YPX media (Table 1).

### Overview of the microarray data

The global transcriptional responses of MA-R4 and MA-B4 to changes in carbon sources, including glucose and xylose, were analyzed by using DNA microarrays at specific time-points during 72 h of fermentation. After the initiation of glucose fermentation using YPD medium, samples of MA-R4 and MA-B4 were harvested at 3 h and 4 h, which were denoted as D1 and D2 stages, respectively (Figure 1A). Samples of MA-R4 and MA-B4 were harvested at 24 h and 48 h (denoted as X1 and *X*2 stages in Figure 1B, respectively) from the start of the cultures containing xylose alone (YPX medium). Thus, these samples were prepared from cells grown until each of the two recombinant *S. cerevisiae* strains had consumed approximately half of each sugar in single-sugar fermentations (glucose or xylose). After the sampling, total RNA was extracted, and then DNA microarray analysis was carried out as described in the Methods section below. For each strain and condition, transcript levels were normalized and quantified by using duplicate (dye-swap) experiments.

**Figure 2:**
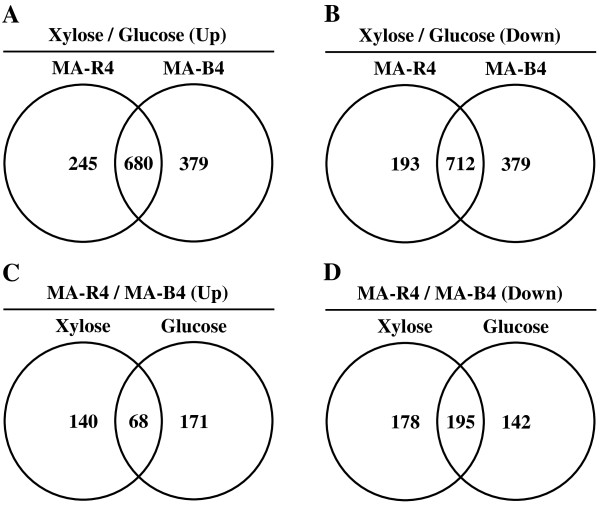
**Venn diagram of common genes with significantly increased expression levels in xylose culture compared to glucose (A), with significantly decreased expression levels in xylose culture compared to glucose (B), with significantly increased expression levels in MA-R4 compared to MA-B4 (C), and with significantly decreased expression levels in MA-R4 compared to MA-B4 (D).** The total number inside each circle represents the number of genes showing a >2-fold change with carbon source or yeast strain in that particular comparison, i.e. 925 up-regulated and 905 down-regulated genes for MA-R4 Xylose/Glucose (C1, Table 2), 1059 up-regulated and 1091 down-regulated genes for MA-B4 Xylose/Glucose (C2, Table 2), 208 up-regulated and 373 down-regulated genes for Xylose MA-R4/MA-B4 (C3, Table 2), and 239 up-regulated and 337 down-regulated genes for Glucose MA-R4/MA-B4 (C4, Table 2).

The microarray data were included in the following four relevant pairwise comparisons of gene expression levels: MA-R4 utilizing xylose vs. MA-R4 utilizing glucose (Comparison 1 = C1); MA-B4 utilizing xylose vs. MA-B4 utilizing glucose (Comparison 2 = C2); MA-R4 utilizing xylose vs. MA-B4 utilizing xylose (Comparison 3 = C3); and MA-R4 utilizing glucose vs. MA-B4 utilizing glucose (Comparison 4 = C4). Next, subsets of genes were identified according to the following criteria: (a) up-regulated genes that changed expression levels on xylose compared with glucose between MA-R4 and MA-B4 (C1 & C2 up, Figure 2A), (b) down-regulated genes that changed expression levels on xylose compared with glucose between MA-R4 and MA-B4 (C1 & C2 down, Figure 2B), (c) up-regulated genes that changed expression levels in MA-R4 compared to MA-B4 on xylose and glucose (C3 & C4 up, Figure 2C), and (d) down-regulated genes that changed expression levels in MA-R4 compared to MA-B4 on xylose and glucose (C3 & C4 down, Figure 2D). A relatively large number of genes, 680 and 712, were up-regulated and down-regulated in both MA-R4 and MA-B4 on xylose compared to glucose, respectively (Figure 2A and B). In contrast, relatively few genes, 68 and 195, were up-regulated and down-regulated on both xylose and glucose in MA-R4 compared to MA-B4, respectively (Figure 2C and D).

### Gene ontology (GO) terms

Using the DNA microarray data obtained, we identified the functional categories of genes with altered expression within the previously described groups (C1, C2, C3, and C4). In general, the identified functional categories would be expected to reflect the functions involved in xylose-utilizing *S. cerevisiae*. In this study, the functions of the gene products were classified by using the gene ontology (GO) terms (http://www.geneontology.org/). Table 2 shows the top ten GO terms (*p* < 0.01) of genes with up-regulated and down-regulated expressions identified within the four pairwise comparisons. If GO terms with multiple synonyms were identified, only the most significant term was listed. In the pairwise comparisons of C1 and C2, many similar GO terms for the up-regulated and down-regulated genes were overrepresented. The GO terms “response to chemical stimulus”, “oxidoreductase activity”, and “generation of precursor metabolites and energy” for the up-regulated genes were overrepresented in both C1 and C2 comparisons. Moreover, the following GO terms for the down-regulated genes were overrepresented in both C1 and C2 comparisons: “intracellular part”, “cytoplasm”, “cellular process”, “intracellular organelle”, “metabolic process”, “cellular metabolic process”, “primary metabolic process”, and “macromolecule metabolic process”. These GO terms include genes involved in the biosynthesis of ribosomes and translation. Interestingly, processes related to amino acid metabolism for the up-regulated genes were overrepresented in the C3 comparison, while processes related to carbohydrate metabolism for the up-regulated genes were overrepresented in the C2 comparison. Increased expression of genes for protein synthesis (in both C1 and C2 comparisons) and amino acid synthesis (in the C3 comparison) appears to correlate directly to the efficiency of anaerobic growth. In addition, the GO terms “oxidoreductase activity” and “lipid metabolic process” were up-regulated by glucose in MA-R4 compared to MA-B4 (C4 comparison). The GO term “lipid metabolic process” may be related to genes involved in ergosterol synthesis, as mentioned in the following subsection. On the other hand, no overrepresented GO terms for down-regulated genes in either C3 or C4 comparisons could be found.

**Table 2:** The top ten gene ontology (GO) terms identified within four pairwise comparisons

**Comparison**	**Regulation**	**GO accession**	**GO term**	***n***	**%**	***p*-value**
MA-R4 xylose/glucose (C1)	Up	GO:0050896	Response to stimulus	174	67.4	5.60E-06
		GO:0006950	Response to stress	123	47.7	6.50E-05
		GO:0042221	Response to chemical stimulus	95	36.8	1.36E-03
		GO:0016491	Oxidoreductase activity	79	30.6	1.14E-06
		GO:0006091	Generation of precursor metabolites and energy	57	22.1	4.38E-05
	Down	GO:0044424	Intracellular part	656	95.8	6.83E-04
		GO:0005737	Cytoplasm	500	73.0	5.16E-06
		GO:0009987	Cellular process	480	70.1	1.99E-06
		GO:0043229	Intracellular organelle	410	59.9	5.18E-03
		GO:0008152	Metabolic process	390	56.9	3.36E-09
		GO:0044237	Cellular metabolic process	390	56.9	1.91E-10
		GO:0044238	Primary metabolic process	374	54.6	3.26E-10
		GO:0043170	Macromolecule metabolic process	321	46.9	1.52E-07
		GO:0043283	Biopolymer metabolic process	321	46.9	7.22E-07
		GO:0006996	Organelle organization	263	38.4	1.94E-08
MA-B4 xylose/glucose (C2)	Up	GO:0042221	Response to chemical stimulus	105	40.9	3.14E-08
		GO:0016491	Oxidoreductase activity	93	36.2	2.78E-08
		GO:0005975	Carbohydrate metabolic process	76	29.6	3.14E-08
		GO:0006091	Generation of precursor metabolites and energy	61	23.7	4.64E-08
	Down	GO:0044424	Intracellular part	852	95.9	1.17E-13
		GO:0044464	Cell part	611	68.8	6.56E-11
		GO:0009987	Cellular process	611	68.8	4.06E-14
		GO:0005737	Cytoplasm	609	68.6	6.14E-06
		GO:0043229	Intracellular organelle	534	60.1	3.13E-05
		GO:0008152	Metabolic process	474	53.4	2.14E-11
		GO:0044237	Cellular metabolic process	474	53.4	8.13E-20
		GO:0044238	Primary metabolic process	457	51.5	4.88E-12
		GO:0005634	Nucleus	404	45.5	2.87E-03
		GO:0043170	Macromolecule metabolic process	382	43.0	1.34E-07
Xylose MA-R4/MA-B4 (C3)	Up	GO:0006520	Cellular amino acid metabolic process	26	100	3.73E-04
	Down	ND	ND	ND	ND	ND
Glucose MA-R4/MA-B4 (C4)	Up	GO:0016491	Oxidoreductase activity	33	61.1	2.54E-05
		GO:0006629	Lipid metabolic process	32	59.3	2.54E-05
	Down	ND	ND	ND	ND	ND

Up- and down-regulated gene ontology (GO) terms identified within four comparisons C1-C4. *n* is the number of the genes with the specified function, % is the percentage of the genes in the comparison with the specified function, and *p*-value is the binomial distribution probability. If no significantly over-represented GO terms were found, this was indicated by “ND” for “not detected”.

### Expression of ergosterol synthesis genes

Sterols like ergosterol are known to play a structural role in membrane integrity, and ergosterol synthesis requires molecular oxygen. We found that the expression of several genes involved in ergosterol synthesis were significantly up-regulated during glucose fermentation in MA-R4 (Table 3). In addition, MA-R4 had higher expression levels of genes related to ergosterol biosynthesis than MA-B4 in fermentation with glucose (Table 3). These results suggest that during glucose fermentation, cellular ergosterol levels in MA-R4 are higher than in MA-B4, and that MA-R4 has a higher ergosterol content in fermentation with glucose than with xylose. These genes encode the enzymes that act both upstream and downstream of ergosterol biosynthesis (*e.g*., *ERG10*, *HMG1*, *ERG1*, and *ERG2*). *ERG10* encodes acetyl-CoA C-acetyltransferase, which catalyzes the first step of sterol biosynthesis [33]. *HMG1* encodes 3-hydroxy-3-methylglutaryl-CoA reductase, which catalyzes the conversion of 3-hydroxy-3-methylglutaryl-CoA to mevalonate, a rate-limiting step of sterol biosynthesis in *S. cerevisiae*[34]. *ERG1* encodes squalene epoxidase, which plays an essential role in the ergosterol biosynthesis pathway [35]. *ERG2* encodes C-8 sterol isomerase, which is responsible for the isomerization of the B ring double bond from the 8 to the 7 position [36]. In MA-R4, *ERG2* was particularly expressed at high levels during glucose fermentation, and its expression level was induced more than 14-fold (Table 3). Thus, the elevated transcription of ergosterol-related genes was specifically seen in MA-R4 during glucose fermentation.

**Table 3:** Expression levels of genes involved in ergosterol biosynthesis

**ORF**	**Gene**	**MA-R4 xylose/glucose (C1)**		**Glucose MA-R4/MA-B4 (C4)**		**Description**
		**(C1-1)**	**(C1-2)**	**(C4-1)**	**(C4-2)**	
YPL028W	*ERG10*	-9.74	-11.19	4.26	3.00	Acetyl-CoA C-acetyltransferase
YML126C	*ERG13*	-9.74	-11.19	4.03	3.65	3-Hydroxy-3-methylglutaryl-CoA synthase
YML075C	*HMG1*	-6.92	-7.35	6.03	6.54	3-Hydroxy-3-methylglutaryl-CoA reductase
YMR208W	*ERG12*	-3.62	-4.78	3.76	3.87	Mevalonate kinase
YMR220W	*ERG8*	-4.50	-3.45	2.11	2.28	Phosphomevalonate kinase
YNR043W	*MVD1*	-9.39	-10.02	2.95	3.52	Mevalonate pyrophosphate decarboxylase
YPL117C	*IDI1*	-2.85	-3.72	2.70	2.54	Isopentenyl diphosphate isomerase
YJL167W	*ERG20*	-6.65	-5.63	3.94	2.73	Farnesyl pyrophosphate synthetase
YHR190W	*ERG1*	-14.58	-9.67	6.22	3.79	Squalene epoxidase
YHR072W	*ERG7*	-5.16	-5.59	2.36	3.24	Lanosterol synthase
YHR007C	*ERG11*	-11.73	-9.61	6.15	4.27	Lanosterol 14-α-demethylase
YGR060W	*ERG25*	-6.56	-7.09	6.17	5.99	C-4 methyl sterol oxidase
YGL001C	*ERG26*	-3.79	-3.71	4.95	4.28	C-3 sterol dehydrogenase
YML008C	*ERG6*	-4.50	-4.41	2.72	2.47	Delta-24-sterol C-methyltransferase
YMR202W	*ERG2*	-21.42	-14.46	4.95	3.73	C-8 sterol isomerase
YLR056W	*ERG3*	-8.00	-9.96	4.17	4.34	C-5 sterol desaturase
YMR015C	*ERG5*	-4.08	-4.95	4.55	6.70	C-22 sterol desaturase

Relevant genes for which the MA-R4 xylose/glucose ratio (C1) was lower than two, and the glucose MA-R4/MA-B4 ratio (C4) was higher than two, were selected. The DNA microarray analysis was repeated twice (Values shown in C1-2 and C4-2 are from dye-swap experiments).

Ergosterol, as well as unsaturated fatty acids, is an essential medium component for anaerobic growth of *S. cerevisiae*[37]. Ergosterol is also important in the ethanol tolerance of yeasts, since deletion of genes in ergosterol biosynthesis prevents proliferation and fermentation of sugars in the presence of moderate ethanol concentrations that are normally tolerated [38]. Taken together, one can assume that the high expression level of ergosterol-related genes in MA-R4 might increase its ability to grow on glucose under anaerobic conditions and/or promote ethanol tolerance. Although these views are speculative at present, they warrant further study. However, oxygen is required for the biosynthesis of ergosterol, and ergosterol cannot be synthesized under anaerobic conditions [39]. That may mean that a shortage of ergosterol under these conditions induces the ergosterol-related genes without enhancing its synthesis. Oxygen stimulates some ergosterol-related genes such as *HMG1* and *ERG9* through the transcriptional factor Hap1p [40,41]. BY4947, the host strain of MA-B4, is a diploid of strain S288C, which carries a Ty1 transposon insertion in the *HAP1* gene. We confirmed no transposon insertion in the *HAP1* gene in IR-2, the host strain for MA-R4 (data not shown). In other words, MA-R4 has wild-type *HAP1* unlike MA-B4, which has a mutant allele of *HAP1*. Therefore, we hypothesized that the higher expression levels of ergosterol-related genes in MA-R4 compared to MA-B4 might be caused by the inactivation of Hap1p in MA-B4. It has been reported that a number of genes encoding enzymes involved in ergosterol biosynthesis are highly expressed in the sake yeast strain Kyokai no. 7 that has wild-type *HAP1*[42]. In any case, more detailed analysis is required to clarify the mechanism by which ergosterol-related genes are induced under these conditions.

### Expression of genes encoding activities of central carbon pathways

**Figure 3:**
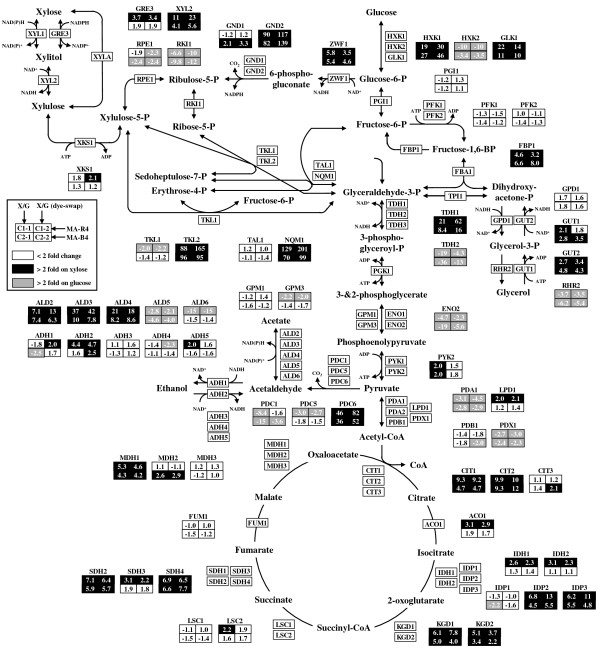
**Expression profiles of genes involved in central carbon metabolism (including glycolysis, PPP, and TCA cycle) of MA-R4 and MA-B4 during fermentation of glucose (G) and xylose (X).** The fold change (log2) calculated as the ratio (xylose/glucose) for transcripts of cells grown on xylose (X1 or *X*2 stages) to cells grown on glucose (D1 or D2 stages) is presented inside of each box for the C1-1, C1-2, C2-1, and C2-2 comparisons; positive values indicate up-regulation and negative values down-regulation on xylose. C1-1 (MA-R4 Xylose/Glucose), C1-2 (MA-R4 Xylose/Glucose, dye-swap), C2-1 (MA-B4 Xylose/Glucose), and C2-2 (MA-B4 Xylose/Glucose, dye-swap) designate specific pairwise comparisons. Transcript levels that changed more than 2-fold with xylose or glucose are shown in black and gray boxes, respectively. Transcript levels that did not change significantly on the two carbon sources are shown in white boxes. The nomenclature follows that of the *Saccharomyces* genome database (http://www.yeastgenome.org/).

To obtain information on genes regulated by a carbon source, expression profiles of genes involved in central carbon metabolism pathways were examined. Figure 3 shows the expression of genes related to central carbon metabolism pathways that consist of glycolysis, gluconeogenesis, the pentose phosphate pathway (PPP), and the tricarboxylic acid (TCA) cycle, altogether leading to the production of metabolic energy. Several reaction steps in central carbon metabolism are irreversible, with more than one isozyme being involved in the catalysis of forward and backward reactions. As expected, there are a large number of genes whose expression is altered by the two carbon sources in both MA-R4 and MA-B4 strains (Figure 3). The hexokinase gene *HXK1* was expressed more highly with xylose than with glucose, regardless of yeast strain (Figure 3). High expression of *HXK1* has previously been reported under aerobic and anaerobic xylose growth [22,25,27], and during growth on non-fermentable carbon sources [43]. The expression of the glucokinase gene *GLK1* is also regulated by non-fermentable carbon sources [43], and was higher with xylose than with glucose in both strains (Figure 3). The *FBP1* gene encoding fructose-1, 6-bisphosphatase 1, the key regulatory enzyme in the gluconeogenesis pathway, was up-regulated during xylose fermentation, irrespective of yeast strain (Figure 3). The *TDH1* gene, which encodes a glyceraldehyde-3-phosphate dehydrogenase (GAPDH) isozyme, is primarily expressed during the stationary phase [44] and was up-regulated with xylose (Figure 3). GAPDH activity is also known to be required during gluconeogenesis. In agreement with these results, when grown on xylose compared to growth on glucose, transcripts for the gluconeogenic enzymes encoded by *ICL1* and *PCK1* were induced 2.3- and 3.7-fold in MA-R4 and 2.1- and 2.8-fold in MA-B4, respectively (data not shown). Thus, the several isozymes specific for gluconeogenesis were induced during anaerobic xylose utilization, which is consistent with previous reports [20,22,25]. On the other hand, transcripts for several enzymes in the lower half of the glycolytic pathway, including some isozymes encoded by *TDH2*, *GPM3*, and *ENO2*, increased during glucose fermentation (Figure 3).

In this study, the heterologous *XYL1* and *XYL2* genes encoding XR and XDH from *S. stipitis* were not included in the *S. cerevisiae* DNA microarrays, but the *S. cerevisiae* genes encoding enzymes with XR (*GRE3*) [45] and XDH (*XYL2*) [46] showed increased expression during fermentation with xylose compared to glucose fermentation (Figure 3). In particular, MA-R4 had a high expression level of *GRE3* and endogenous *XYL2* during fermentation with xylose. In MA-R4, the *XKS1* gene encoding XK was expressed at levels about twice as high when xylose was the only sugar as when glucose was the only sugar (Figure 3). Interestingly, *XKS1* was more highly expressed in MA-R4 than in MA-B4 under both fermentation conditions (Table 4).

**Table 4:** Expression levels of genes significantly increased in MA-R4 relative to MA-B4

**ORF**	**Gene**	**Xylose MA-R4/MA-B4 (C3)**		**Glucose MA-R4/MA-B4 (C4)**		**Description**
		**(C3-1)**	**(C3-2)**	**(C4-1)**	**(C4-2)**	
Xylose metabolism						
YGR194C	*XKS1*	7.23	6.13	11.97	9.20	Xylulokinase
Hexose transporters						
YMR011W	*HXT2*	41.40	26.68	2.20	2.38	High-affinity glucose transporter
YDR345C	*HXT8*	7.17	16.25	4.10	5.35	Protein of unknown function with similarity to hexose transporter family members
Other transporters						
YCR098C	*GIT1*	13.32	8.90	2.53	2.28	Plasma membrane permease, mediates uptake of glycerophosphoinositol and glycerophosphocholine
YLR237W	*THI7*	2.33	2.97	2.05	2.14	Plasma membrane transporter responsible for the uptake of thiamine
YKR093W	*PTR2*	2.32	2.69	8.30	8.87	Integral membrane peptide transporter
ATP synthesis						
YBR085W	*AAC3*	3.42	3.08	4.89	3.68	Mitochondrial inner membrane ADP/ATP translocator
Q0080	*ATP8*	4.25	5.61	3.65	4.33	Subunit 8 of the F0 sector of mitochondrial inner membrane F1-F0 ATP synthase

Relevant genes for which the xylose MA-R4/MA-B4 ratio (C3) was higher than two, and the glucose MA-R4/MA-B4 ratio (C4) was higher than two, were selected. The DNA microarray analysis was repeated twice (Values shown in C3-2 and C4-2 are from dye-swap experiments).

In the oxidative part of the PPP, the *ZWF1* and *GND2* genes, encoding glucose-6-phosphate dehydrogenase and NADPH-producing 6-phosphogluconate dehydrogenase, respectively, were significantly up-regulated with xylose in MA-R4 and MA-B4 (Figure 3). In particular, *GND2* was highly increased by more than 80-fold in fermentation with xylose compared to glucose. These results support the idea that low carbon flux through glycolysis from the PPP is one of the biggest factors restricting xylose utilization [30]. It is also worth noting that *ZWF1* and *GND2* are induced during the stress response, perhaps to help replenish NADPH reducing equivalents [47,48]. The *NQM1* and *TKL2* genes, encoding the minor transaldolase and transketolase in the non-oxidative PPP [49], were significantly up-regulated during xylose fermentation in both strains, and their expression levels were increased more than 70-fold and 88-fold, respectively (Figure 3). These results are consistent with our previous finding that transcription of *NQM1* in MA-R4 is elevated more than 4-fold in xylose-containing medium compared with that in glucose-containing medium by real-time PCR [49]. Therefore, these results strongly support the view that *NQM1* may be involved in the xylose metabolic pathway or in an unknown xylose utilizing pathway via the non-oxidative PPP [49]. Meanwhile, it was unexpected that in MA-R4 and MA-B4, the transcript levels of *TKL2* would be greatly elevated in the xylose-containing medium compared with glucose-containing medium (Figure 3 and Table 5), because our previous studies showed that the expression of *TKL2* is very low in MA-R4 regardless of the culture conditions [49]. *TKL2* is induced in response to heat shock in an Msn2/4p-dependent manner [50], and several target genes of Msn2/4p, including *TKL2*, were greatly up-regulated with xylose (Table 5). Taking these results together, it is tempting to speculate that the induction of *TKL2* was increased through Msn2/4p-mediated stress responses rather than in response to xylose as the sole carbon source. On the other hand, the transcription of *TAL1* and *TKL1*, the major transaldolase and transketolase genes, was relatively unchanged between xylose-grown and glucose-grown cells (Figure 3). Other genes encoding enzymes of the non-oxidative PPP, ribulose-5-phosphate 3-epimerase (*RPE1*) and ribose-5-phosphate ketol-isomerase (*RKI1*), were expressed at lower levels in yeast cells cultivated on xylose (Figure 3).

**Table 5:** Expression levels of known Msn2/4p-dependent genes

**ORF**	**Gene**	**MA-R4 xylose/glucose (C1)**		**MA-B4 xylose/glucose (C2)**		**Description**
		**(C1-1)**	**(C1-2)**	**(C2-1)**	**(C2-2)**	
YER150W	*SPI1*	126.64	199.67	48.44	67.29	GPI-anchored cell wall protein
YOL052C-A	*DDR2*	105.36	135.30	99.99	232.51	Multi-stress response protein
YBR117C	*TKL2*	87.83	164.53	95.81	94.69	Transketolase
YGR088W	*CTT1*	38.28	49.52	17.11	21.33	Catalase T
YMR169C	*ALD3*	37.09	42.34	10.28	7.78	Aldehyde dehydrogenase
YBL075C	*SSA3*	24.17	17.42	12.94	14.98	Chaperone protein
YLR258W	*GSY2*	11.84	16.29	5.01	6.14	Glycogen synthase
YDR171W	*HSP42*	10.65	9.66	5.25	5.53	Heat shock protein
YDR258C	*HSP78*	10.11	7.85	5.36	4.66	Heat shock protein
YMR170C	*ALD2*	7.08	12.90	7.38	6.28	Aldehyde dehydrogenase
YLL026W	*HSP104*	6.89	7.95	6.11	6.61	Heat shock protein
YDR074W	*TPS2*	5.27	4.61	4.55	4.72	Trehalose-6-phosphate phosphatase
YBR126C	*TPS1*	4.95	6.86	4.75	7.17	Trehalose-6-phosphate synthase
YHR008C	*SOD2*	2.48	2.14	2.65	2.29	Superoxide dismutase

Relevant genes for which the MA-R4 xylose/glucose ratio (C1) and the MA-B4 xylose/glucose ratio (C2) were higher than two were selected. The DNA microarray analysis was repeated twice (Values shown in C1-2 and C2-2 are from dye-swap experiments).

Several genes encoding TCA cycle enzymes increased in xylose-utilizing yeast strains, and many of them (*e.g. CIT1*, *CIT2*, *IDP2*, *IDP3*, *KGD1*, *KGD2*, *SDH2*, *SDH4*, and *MDH1*) were induced to a greater extent in MA-R4 compared with MA-B4 (Figure 3). There were some exceptions with regard to the expression of the genes; *MDH2*, which encodes cytosolic malate dehydrogenase, was expressed at a relatively high level only in MA-B4, and the expressions of the mitochondrial NADP^+^-dependent isocitrate dehydrogenase gene *IDP1*, the succinyl-CoA ligase gene *LSC1*, and the fumarase gene *FUM1* were slightly lower with xylose in both strains. In addition to the fact that TCA cycle genes were expressed more strongly in xylose-utilizing cells than in glucose-utilizing cells, genes involved in respiration (*e.g. COR1*, *COX1*, *COX5B*, *QCR8*, *CYC3*, and *CYC7*) were up-regulated during xylose fermentation (Table 6). Thus, the expression of genes encoding the TCA cycle and respiration enzymes increased significantly when strains were cultivated anaerobically on xylose, indicating that *S. cerevisiae* engineered for xylose metabolism does not exhibit a fermentative response to the sugar even under anaerobic conditions. Induced transcription of genes encoding enzymes of the TCA cycle and respiration during xylose metabolism has also been observed in previous studies [22,25,27]. Respiratory conditions increase reactive oxygen species (ROS), so that genes involved in adaptation to ROS, such as *MGA2* that is implicated in DNA repair [51], should also increase. In the present study *MGA2* in both strains increased by more than 3-fold in xylose-containing medium compared with glucose-containing medium (data not shown), indicating that xylose induces adaptation to oxidative stress. It should be also noted that in MA-B4, transcript levels of *HAP4*, encoding a transcriptional activator and global regulator of respiratory gene expression [52], were more than 6.7-fold higher with xylose than with glucose, but did not change significantly with respect to carbon sources in MA-R4 (data not shown). It is also worth mentioning that stress responses induce the expression of genes that are implicated in respiration, including *CIT1* and *CYC7*. Increasing the levels of these gene products may promote ATP synthesis by utilizing existing respiration components. Alternatively, the induction of factors that promote cytochrome *c* and ubiquinone synthesis may play a role in the defense against oxidative stress rather than in ATP generation [53].

**Table 6:** Expression levels of genes with significantly changed in xylose metabolism relative to glucose metabolism

**ORF**	**Gene**	**MA-R4 xylose/glucose (C1)**		**MA-B4 xylose/glucose (C2)**		**Description**
		**(C1-1)**	**(C1-2)**	**(C2-1)**	**(C2-2)**	
Respiratory metabolism						
YBL045C	*COR1*	2.37	2.73	3.00	2.75	Core subunit of the ubiquinol-cytochrome *c* reductase complex (bc1 complex)
Q0045	*COX1*	2.17	2.55	2.25	2.50	Subunit I of cytochrome *c* oxidase
YIL111W	*COX5B*	7.20	11.74	2.87	2.62	Subunit Vb of cytochrome c oxidase
YMR256C	*COX7*	2.06	2.38	3.87	3.95	Subunit VII of cytochrome *c* oxidase
YGL191W	*COX13*	2.15	2.89	4.21	5.33	Subunit VIa of cytochrome *c* oxidase
YDR231C	*COX20*	4.97	4.39	4.61	4.94	Mitochondrial inner membrane protein
YJL166W	*QCR8*	2.66	3.36	3.87	5.50	Subunit 8 of ubiquinol cytochrome *c* reductase complex
YAL039C	*CYC3*	2.31	2.11	5.63	6.08	Cytochrome *c* heme lyase (holocytochrome *c* synthase)
YEL039C	*CYC7*	7.40	8.71	31.54	37.06	Cytochrome c isoform 2
YPL159C	*PET20*	2.54	2.98	2.13	2.80	Mitochondrial protein
YLR154W-C	*TAR1*	2.25	4.70	2.32	4.56	Mitochondrial protein
Hexose transporters						
YHR094C	*HXT1*	-30.45	-21.51	-56.36	-46.31	Low-affinity glucose transporter
YDR345C	*HXT3*	-54.60	-20.87	-83.61	-32.66	Low-affinity glucose transporter
YHR096C	*HXT5*	22.20	48.69	22.78	44.86	Hexose transporter with moderate affinity for glucose
Galactose metabolism						
YBR020W	*GAL1*	19.10	32.47	3.71	5.48	Galactokinase
YDR009W	*GAL3*	4.20	4.91	6.88	9.85	Transcriptional regulator involved in activation of the *GAL* genes in response to galactose
YPL248C	*GAL4*	3.44	4.04	9.38	7.75	DNA-binding transcription factor required for the activation of the *GAL* genes in response to galactose
YBR018C	*GAL7*	7.05	13.98	5.29	10.70	Galactose-1-phosphate uridyl transferase
YBR019C	*GAL10*	45.93	203.38	16.32	14.96	UDP-glucose-4-epimerase
Spore wall metabolism						
YHR139C	*SPS100*	53.16	93.57	12.99	18.00	Protein required for spore wall maturation
YDR403W	*DIT1*	4.77	8.66	4.68	6.11	Sporulation-specific enzyme required for spore wall maturation
YDR402C	*DIT2*	3.86	2.59	6.18	3.33	N-formyltyrosine oxidase
YGR032W	*GSC2*	10.25	15.12	9.91	17.89	Catalytic subunit of 1,3-beta-glucan synthase
YMR306W	*FKS3*	8.57	9.05	10.66	7.54	Protein involved in spore wall assembly
YDL239C	*ADY3*	2.89	3.82	2.31	2.26	Protein required for spore wall formation
Ammonia secretion						
YNR002C	*ATO2*	8.78	36.19	9.57	13.26	Putative transmembrane protein involved in export of ammonia
Trehalose metabolism						
YBR126C	*TPS1*	4.95	6.86	4.75	7.17	Synthase subunit of trehalose-6-P synthase/phosphatase complex
YDR074W	*TPS2*	5.27	4.61	4.55	4.72	Phosphatase subunit of the trehalose-6-P synthase/phosphatase complex
YML100W	*TSL1*	17.88	18.11	8.01	6.82	Large subunit of trehalose 6-phosphate synthase/phosphatase complex

Relevant genes for which the MA-R4 xylose/glucose ratio (C1) was lower than two, and the MA-B4 xylose/glucose ratio (C2) was higher than two, were selected. The DNA microarray analysis was repeated twice (Values shown in C1-2 and C2-2 are from dye-swap experiments).

In the alcohol catabolism and by-product pathways, several genes were changed significantly. The major isozymes of the pyruvate decarboxylase gene *PDC1* were up-regulated with glucose, while *PDC6*, encoding the minor isoform of pyruvate decarboxylase, was highly up-regulated with xylose in both MA-R4 and MA-B4 (Figure 3). Interestingly, *PDC6* is strongly induced under nutrient-limited conditions, especially during sulfur deficiency [54]. Transcripts of *ADH2*, which encodes glucose-repressible alcohol dehydrogenase 2, increased with xylose, especially in MA-R4 (Figure 3). In general, *ADH2* as well as *HXK1* are repressed by glucose. The genes encoding the transcription factors *ADR1* and *CAT8*, which regulate the expression of *ADH2*[55,56], had more than 10-fold higher transcript levels with xylose than with glucose in both strains (data not shown). Another ethanol oxidation enzyme, acetyl-CoA synthetase encoded by *ACS1*, is also regulated by Adr1p and Cat8p [57], but its expression did not change significantly between the two carbon sources (data not shown). Among the *ALD* genes involved in acetate formation, isoenzymes of cytosolic aldehyde dehydrogenases, encoded by *ALD2* and *ALD3*, and the mitochondrial aldehyde dehydrogenase gene *ALD4*, were up-regulated with xylose (Figure 3), suggesting that acetaldehyde was utilized for growth instead of ethanol production during xylose fermentation. In contrast, the expression of *ALD5*, encoding another mitochondrial aldehyde dehydrogenase, was lower with xylose (Figure 3). Meanwhile, in only MA-R4, *ALD6*, which is responsible for the cytoplasmic synthesis of acetate from acetaldehyde, had its lowest abundance with xylose (Figure 3). The expression of glucose-repressible *ACH1*, encoding a mitochondrial acetyl-CoA hydrolase [58], had more than 9-fold higher transcription with xylose than with glucose in both strains (data not shown). The observed higher expression of genes involved in acetaldehyde and acetyl-CoA metabolism during xylose fermentation is consistent with a previous report [25] suggesting that the mitochondrial acetate concentration is perhaps elevated during growth on xylose compared with growth on glucose. In the glycerol catabolism pathway, the expression of genes *GUT1* and *GUT2*, encoding glycerol kinase and mitochondrial glycerol 3-phosphate dehydrogenase, respectively, increased during xylose fermentation (Figure 3). Conversely, the glycerol-producing gene *RHR2*, encoding glycerol 1-phosphatase, had a lower expression level with xylose than with glucose (Figure 3). The expression of *GUT1* and *GUT2* together with *HXK1*, *GLK1*, *FBP1*, and *PCK1* is known to increase during growth on non-fermentable carbon sources [43,59,60]. Combining these results, we can conclude that xylose-utilizing *S. cerevisiae* recognizes xylose as a non-fermentable carbon source.

### Expression of sugar transporter genes and galactose metabolism genes

Although hexose transporters (encoded by the *HXT* genes) in *S. cerevisiae* transport not only glucose but also xylose, these transporters have less specificity and lower affinity for xylose than for glucose [61]. We confirmed that transcript levels of *HXT1* and *HXT3* encoding the low-affinity glucose transporters were highly induced in the strains grown on glucose (Table 6), which is consistent with a previous finding that the expression of *HXT1* and *HXT3* is induced by high glucose concentrations [62]. In contrast, the *HXT5* gene, encoding a functional hexose transporter that has moderate affinity for glucose (40 mM) [63], had higher expression in fermentation with xylose compared with glucose (Table 6), suggesting that glucose represses the transcription of *HXT5*. Transcription of *HXT5* is induced in the presence of non-fermentable carbon sources [63] and during carbon source limitation [54]. Therefore, these results support the idea that xylose is sensed as a non-fermentable carbon source, and the expression of *HXT5* might be induced under conditions of energy and carbon source limitation. In xylose-containing medium, there was also an increase in the expression of a gene implicated as being a hexose uptake sensor (*SNF3*), which showed more than 3- and 5-fold higher transcription in MA-R4 and MA-B4, respectively (data not shown). *HXT2*, encoding a low-affinity glucose transporter, and *HXT8*, encoding a glucose transporter-like protein, had higher transcript levels in MA-R4 than in MA-B4 (Table 4). The expression of these two genes is induced by low levels of glucose and repressed by high levels of glucose [64].

Together with some hexose transport genes, the gene encoding galactose permease (*GAL2*) was up-regulated by more than 7.6-fold with xylose in both MA-R4 and MA-B4 (data not shown). *GAL2* has been shown to transport xylose as well as galactose and glucose [61], although overexpressing *GAL2* does not improve xylose growth [65]. In addition, *GAL1*, *GAL3*, *GAL4*, *GAL7*, and *GAL10* in galactose metabolism were up-regulated by xylose (Table 6). The activation of galactose metabolism-related genes is induced by the dominant positive regulator coding gene *GAL3*, and the higher expression level of *GAL3* (Table 6) probably resulted in the strong up-regulation of *GAL* genes [23,66]. Our finding that the expression of *GAL* genes was higher when xylose was the only sugar than when glucose was the only sugar is consistent with other published DNA microarray data [23,65,66]. In addition, the *MIG1* gene, encoding a transcription factor involved in glucose repression, also regulates genes involved in galactose metabolism [20]. In the presence of glucose, *MIG1* causes repression of the transcription of genes implicated in galactose metabolism [67]. It is worth mentioning that transcription of *MIG1* was induced more than 3.7-fold in MA-R4 when grown on glucose compared to xylose, although the expression of *MIG1* was comparable in the glucose- and xylose-grown MA-B4 (data not shown). Thus, the results suggest that in MA-R4, induction of galactose metabolism-related genes by xylose is partially regulated by Mig1p.

### Starvation response during xylose fermentation

The absence of a nitrogen source combined with the presence of a non-fermentable carbon source leads diploid cells of *S. cerevisiae* to enter the developmental pathway of meiosis and sporulation [68]. In both xylose-fermenting diploid strains of *S. cerevisiae*, MA-R4 and MA-B4, several genes involved in spore wall metabolism (*SPS100*, *DIT1*, *DIT2*, *GSC2*, *FKS3*, and *ADY3*) showed an increase in expression during xylose fermentation (Table 6). *SPS100*, which contributes to spore wall maturation [69], was dramatically induced in the presence of xylose, especially in MA-R4 (Table 6). *DIT1* and *DIT2*, which encode enzymes mediating spore wall assembly [70]; *GSC2* and *FKS3*, which are involved in the formation of the inner (β-glucan) layer of the spore wall [71]; and *ADY3*, which encodes a protein required for spore wall formation [72], were also up-regulated in cells grown on xylose (Table 6).

In addition to the genes involved in spore wall metabolism, the expression of *ATO2* encoding a putative ammonium transporter had higher transcript levels with xylose in the two recombinant yeast strains (Table 6). Ato2p, together with Ato1p and Ato3p, are all members of the YaaH family of proteins with six transmembrane domains that has been proposed to be involved in the export of ammonia, and their genes were strongly expressed during the alkali phase of colony growth that is accompanied by increased ammonia production [73]. The ammonia release is a starvation signal that directs the growth of colonies away from neighboring colonies and toward more nutrient-rich areas on the plate [24,73]. However, the transcript levels of another putative ammonium transporter gene, *ATO3*, did not change significantly with respect to the carbon source in MA-R4 and MA-B4 (data not shown). In addition, *ATO1* was not detected in this study.

### Expression of Msn2/4p-mediated genes

Msn2p and Msn4p (Msn2/4p) are functionally redundant transcription factors that regulate the general stress response of *S. cerevisiae*[74,75]. Each factor binds to a heat shock element (HSE) or stress response element (STRE) found in the promoters of many stress response genes. The known target genes of Msn2/4p were remarkably up-regulated with xylose in both MA-R4 and MA-B4 (Table 5). Genes up-regulated in the presence of xylose included the following large set of stress-responsive genes: *SPI1*, which encodes a member of the glycosylphosphatidylinositol (GPI)-anchored cell wall protein family that protects the yeast cell from damage caused by weak acids [76]; *DDR2*, which encodes a multi-stress response protein and is transcriptionally activated by a variety of xenobiotic agents and environmental or physiological stresses [77]; carbohydrate metabolism-related genes (*TKL2*, *ALD2*, *ALD3*, *GSY2*, *TPS1*, and *TPS2*); oxidative stress defense-related genes (*CTT1* and *SOD2*); and protein folding chaperones (*SSA3*, *HSP42*, *HSP78*, and *HSP104*). Among these stress-induced genes, *SPI1* was highly increased by more than 126-fold in MA-R4 when fermenting with xylose compared to glucose (Table 5). *DDR2* was also significantly up-regulated during xylose fermentation in both MA-R4 and MA-B4, and its transcript levels were induced more than 105-fold and 99-fold, respectively (Table 5). In agreement with the elevated expression levels of a large number of Msn2/4p gene targets in the xylose culture, the transcription of *MSN4* (data not shown) was induced during xylose fermentation more than 2.7- and 4.4-fold in MA-R4 and MA-B4, respectively. However, the levels of *MSN2* transcripts were comparable in xylose and glucose cultures (data not shown). As with many other Msn2/4p-mediated genes containing STREs, the expression of the trehalose synthase genes, *TPS1*, *TPS2*, and *TSL1*, was also induced in the presence of xylose (Table 6). It has been shown that transcripts of these three genes are coregulated and increase under various stress conditions, including nutrient starvation [78,79], and that trehalose serve as a stress protectant in many organisms.

At present, the molecular mechanism of the regulation of stress-responsive genes in response to xylose remains elusive, but the results of the present study strongly support the view that genes that respond to starvation for nutrients, such as carbon and nitrogen sources, and oxidative stress are induced during xylose fermentation. This hypothesis was confirmed by the finding that several genes involved in spore wall metabolism (*SPS100*, *DIT1*, *DIT2*, *GSC2*, *FKS3*, and *ADY3*), the putative ammonium transporter gene encoded by *ATO2*, and the minor isoform of pyruvate decarboxylase gene *PDC6*, as well as a number of TCA cycle and respiration genes, increased in expression when fermenting with xylose (Table 6 and Figure 3). In our previous paper [30], we also showed that carbon and energy starvation conditions are normal in MA-R4 during fermentation with xylose. Therefore, it is also tempting to speculate that recombinant xylose-metabolizing *S. cerevisiae* strains respond to xylose as though they were experiencing carbon and energy starvation, leading to the activation of Msn2/4p-dependent transcription.

### Expression of genes increased in MA-R4 relative to MA-B4

Recombinant industrial *S. cerevisiae* strains such as MA-R4 have some special features for ethanol production, including high ethanol productivity, high tolerance to ethanol, and tolerance to inhibitory compounds, compared to recombinant laboratory strains such as MA-B4 [3,31]. From gene expression profiles, we obtained further evidence for these special features and other abilities of MA-R4. As mentioned above, two hexose transport genes, *HXT2* and *HXT8*, as well as the *XKS1* gene encoding xylulokinase (XK) in xylose metabolism had higher transcript levels in MA-R4 than in MA-B4, irrespective of carbon source (Table 4). The higher expression levels of *XKS1* found in MA-R4 (Table 4) agreed well with the higher XK activities (more than doubled) in the cell extracts (data not shown). Some genes encoding membrane transporters other than sugar transporters (*GIT1*, *THI7*, and *PTR2*) were also more highly expressed in MA-R4 than in MA-B4 (Table 4). *GIT1* encodes a permease involved in the uptake of glycerophosphoinositol (GroPIns) and glycerophosphocholine (GroPCho) as sources of the nutrients inositol and phosphate [80,81]. Inositol and phosphate are important nutrients for all eukaryotic cells, including *S. cerevisiae*; inositol serves as an important component of the structural phosphatidylinositol class of lipids, and phosphate is required to make two of the most important organic macromolecules, DNA and ATP. It is known that much of the GroPIns produced are excreted into the medium, and external GroPIns can be transported into yeast cells in times of nutritional stress (inositol or phosphate limitation) via Git1p permease [80]. Therefore, it is reasonable to hypothesize that during fermentation, MA-R4 can transport and utilize inositol and/or phosphate more efficiently compared with MA-B4 in response to these nutritional stresses. Meanwhile, *THI7* encodes a transporter responsible for the uptake of thiamine [82]. Thiamine is an important co-factor of pyruvate decarboxylase for ethanol production in yeast cells. Therefore, one can assume that the level of thiamine in MA-R4 might be low due to the consumption of thiamine in yeast cells as a co-factor for pyruvate decarboxylase in ethanol fermentation. *PTR2* encodes an integral membrane peptide transporter that mediates the transport of di- and tri-peptides [83]. Utilization of di/tripeptides as nitrogen and carbon sources and for protein synthesis is an important cellular process in all organisms, and di/tri-peptides regulate a variety of cellular processes such as gene transcription, protein translation, and enzyme activity. In yeasts, peptide transport is up-regulated in growth media containing poor nitrogen sources. Interestingly, *GIT1* showed high expression levels during xylose fermentation, whereas *PTR2* had high expression levels during glucose fermentation (Table 4). Taken together, the uptake of GroPIns, GroPCho, thiamine, and di/tripeptides is important for the higher rates of anaerobic growth and sugar consumption, and the higher fermentation ability, of MA-R4 as a recombinant industrial yeast. It would also be of interest to examine the intracellular levels of these substrates in MA-R4 and MA-B4 during fermentation.

The ADP/ATP translocator gene *AAC3*[84] and ATP synthase gene *ATP8*[85], both of which are regulated at the mitochondrial inner membrane to generate ATP from ADP, were up-regulated in MA-R4 regardless of carbon source (Table 4), implying that MA-R4 gains more energy for the cell through ATP synthesis than MA-B4. It has been suggested that the rate of ATP formation is the primary limiting factor for anaerobic growth on xylose alone [23]. Therefore, lower ATP levels in MA-B4 compared with MA-R4 may be responsible for the lower efficiency of anaerobic growth and fermentation of MA-B4. Interestingly, the deletion of *ATP8*, like deletions in many genes necessary for the function or maintenance of mitochondria, leads to a “petite” phenotype that is slow-growing and unable to survive on non-fermentable carbon sources [86]. Thus, genes involved in ATP synthesis as well as genes encoding a variety of membrane transporters were expressed at higher levels in MA-R4 than in MA-B4 regardless of the culture conditions used in this study.

## Conclusions

The results of this study using genome-wide gene expression analysis strongly support the conclusion that MA-R4 acquires more energy and nutrients from its higher fermentation ability via the uptake of different compounds and the synthesis of ATP compared to MA-B4, and that high expression levels of ergosterol-related genes in MA-R4 during glucose fermentation may increase its ability to grow on glucose under anaerobic conditions and/or its ethanol tolerance. This study has further demonstrated that xylose-utilizing *S. cerevisiae* senses xylose as a non-fermentable carbon source, which then induces a starvation response and increases ROS, leading to an increase in the expression of many genes involved in stress. These views are still speculative, and none of the above assumptions has yet been fully proven. However, these ideas provide us with a preliminary basis for understanding the molecular events underlying the response to xylose or glucose, and the differences between industrial and laboratory strains during fermentation.

## Methods

### Yeast strains and media

The recombinant xylose-utilizing *S. cerevisiae* strains MA-R4 and MA-B4 were used in this study. MA-R4, derived from the industrial diploid and flocculent yeast strain IR-2, was genetically engineered with the chromosome-integrated *XYL1* and *XYL2* genes that encode XR and XDH from *S. stipitis*, along with the endogenous *XKS1* gene that encodes XK under the control of the *PGK* promoter [31,87]. *S. cerevisiae* laboratory strain BY4947 (diploid of S288C, *MAT*a/α *SUC2/SUC2 mal/mal mel/mel gal2/gal2 CUP1/CUP1 flo1/flo1 flo8-1/flo8-1 SSD1-v1*/*SSD1-v1*), which was obtained from the Yeast Genetic Resource Center (YGRC, Osaka University, Osaka, Japan), was another recipient yeast strain for the expression of *XYL1*, *XYL2*, and *XKS1* genes. For the construction of strains MA-R4 and MA-B4, plasmid pAUR-XKXDHXR [88] was digested with the restriction enzyme *Bsi*WI and chromosomally integrated into the *aur1* locus of IR-2 and BY4947, respectively. MA-R4 and MA-B4 were maintained by selective growth on yeast peptone (YP) medium (10 g/L yeast extract and 20 g/L peptone) supplemented with 20 g/L glucose in the presence of 0.5 mg/L aureobasidin A (Takara Bio, Kyoto, Japan). Glucose (40 g/L) was added to YP medium to produce YPD medium. Xylose (40 g/L) was added to YP medium to produce YPX medium. The YPD and YPX media were used as the anaerobic fermentation media in this study.

### Fermentation

For anaerobic batch fermentation, MA-R4 and MA-B4 were first cultivated aerobically in 5 mL YP medium supplemented with 20 g/L glucose and 0.5 mg/L aureobasidin A for 36 h at 30°C. The resulting cultures were centrifuged at 6000 × *g* for 5 min at 4°C, and the pelleted cells were washed and resuspended in distilled water. The washed cells were inoculated into 20 mL fermentation medium (YPD and YPX) in which aureobasidin A was not included. For all fermentation media, the initial cell density was adjusted to approximately 2.34 g (dry cell weight (DCW)) per liter. Anaerobic batch fermentations were performed at 30°C in 50-mL sterilized closed bottles with magnetic stirring as described previously [31,87,88]. Samples (0.3 mL) of fermentation broth (YPD and YPX) were taken at specified intervals and diluted 4-fold with 8 mM H_2_SO_4_. These diluted samples were stored at -30°C for high-performance liquid chromatography (HPLC) analysis of substrates and fermentation products. All experiments were performed in triplicate.

### Quantification of biomass, substrates, and fermentation products

DCW was determined using a UV-2450 spectrophotometer (Shimadzu, Kyoto, Japan) to measure the absorbance of the samples at 600 nm, as described previously [88]. The maximum specific production rates of ethanol and consumption rates of glucose and xylose were calculated as the slope in linear regression of metabolite concentration divided by DCW vs. time [89]. Concentrations of glucose, xylose, ethanol, xylitol, glycerol, and acetic acid were determined with an HPLC apparatus (Jasco, Tokyo, Japan) equipped with a refractive index detector (RI-2031Plus; Jasco) using an Aminex HPX-87H (Bio-Rad Laboratories, Hercules, CA, USA) and Cation H Refill Guard (Bio-Rad) column. The HPLC apparatus was operated at 65°C, with 5 mM H_2_SO_4_ as the mobile phase, a flow rate of 0.6 mL/min, and an injection volume of 20 μL.

### Extraction of RNA

Yeast cells grown as described above were harvested for transcriptional analysis at times indicated in the Results and Discussion section above. Duplicate samples (approximately 6.44 g/L DCW) were collected at each time point (all four time points). Total RNA was isolated using the FastRNA Pro Red Kit (Q-BIOgene, Irvine, CA, USA), according to the manufacturer’s protocols. RNA was further purified using the RNeasy Mini Kit (Qiagen, Hilden, Germany). RNA quality and concentration were measured using an Agilent 2100 Bioanalyzer (Agilent Technologies, Palo Alto, CA, USA) and NanoDrop ND-1000 (Thermo Fisher Scientific, Waltham, MA, USA), respectively.

### DNA microarray analysis and data processing

DNA microarray analysis was performed using the 3D-Gene Yeast Oligo Chip 6 k (Toray Industries Inc., Tokyo, Japan/DNA Chip Research, Inc., Yokohama, Japan) as described previously [90-92]. Using total RNA isolated as described above, the amplification and preparation of amino allyl RNA (aRNA) and the labeling of aRNA with the fluorescence dyes Cy3 and Cy5 were carried out with the Amino Allyl MessageAMP II aRNA Amplification Kit (Applied Biosystems, CA, USA). The Cy3- or Cy5-labeled aRNA pools and hybridization buffer containing micro beads were mixed on the 3D-Gene Yeast Oligo Chip 6 k for 16 h at 37°C. This chip has 3-dimensions that is constructed with a well as the space between the probes and cylinder-stems with 30-mer oligonucleotide probes on the top for efficient hybridization of the Cy3- or Cy5-labeled aRNAs and the probes, and contains all 5795 yeast genes registered in the *Saccharomyces* Genome Database (SGD). The hybridization and wash steps were performed following the supplier’s protocols. The hybridized DNA chip was scanned using ScanArray Express HT (PerkinElmer, Waltham, MA, USA). The normalization of the fluorescence intensities of each probe between Cy3 and Cy5 was achieved by the intensity dependent (LOWESS) methods [93]. The cutoff values were defined by the intensity of the background average plus 2SD. Data processing, including hierarchical cluster analysis, was performed using GeneSpringGX10 software (Agilent Technologies). We further used a dye-swap experiment (also called color flip experiment) to show that the dye used for labeling does not cause any bias in allele frequency measurement.

## Abbreviations

CoA: Coenzyme A; DCW: Dry cell weight; GO: Gene ontology; GPI: Glycosylphosphatidylinositol; GroPCho: Glycerophosphocholine; GroPIns: Glycerophosphoinositol; HPLC: High-performance liquid chromatography; P: Phosphate; PPP: Pentose phosphate pathway; ROS: Reactive oxygen species; TCA: Tricarboxylic acid; XDH: Xylitol dehydrogenase; XI: Xylose isomerase; XK: Xylulokinase; XR: Xylose reductase; YP: Yeast peptone.

## Competing interests

The authors declare that they have no competing interests.

## Authors’ contributions

AM participated in the design of the study, performed experimental work, and wrote the manuscript. TG performed the batch fermentations. TH participated in the design of the study and commented on the manuscript. All authors have read and approved the final manuscript.
